# Sex differences in longitudinal personality stability in chimpanzees

**DOI:** 10.1017/ehs.2020.45

**Published:** 2020-09-09

**Authors:** Bruce Rawlings, Emma Flynn, Hani Freeman, Lisa Reamer, Steven J Schapiro, Susan Lambeth, Rachel L Kendal

**Affiliations:** 1 Durham Cultural Evolution Research Centre, Department of Anthropology, Durham University, UK; 2 Department of Psychology, University of Texas at Austin, TX, USA; 3 National Center for Chimpanzee Care, Michale E. Keeling Center for Comparative Medicine and Research, The University of Texas MD Anderson Cancer Center, Bastrop, TX, USA; 4 School of Psychology, Queen's University, Belfast, UK; 5 Department of Experimental Medicine, University of Copenhagen, Copenhagen, Denmark

**Keywords:** Chimpanzees, personality, longitudinal, sex-differences

## Abstract

Personality factors analogous to the Big Five observed in humans are present in the great apes. However, few studies have examined the long-term stability of great ape personality, particularly using factor-based personality instruments. Here, we assessed overall group, and individual-level, stability of chimpanzee personality by collecting ratings for chimpanzees (*N* = 50) and comparing them with ratings collected approximately 10 years previously, using the same personality scale. The overall mean scores of three of the six factors differed across the two time points. Sex differences in personality were also observed, with overall sex differences found for three traits, and males and females showing different trajectories for two further traits over the 10 year period. Regardless of sex, rank-order stability analysis revealed strong stability for dominance; individuals who were dominant at the first time point were also dominant 10 years later. The other personality factors exhibited poor to moderate rank-order stability, indicating that individuals were variable in their rank-position consistency over time. As many studies assessing chimpanzee cognition rely on personality data collected several years prior to testing, these data highlight the importance of collecting current personality data when correlating them with cognitive performance.

## Graphical abstract

**Figure:**
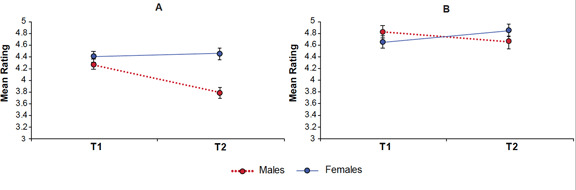

**Media summary:** This study assessed male and female chimpanzee personality stability over a decade and how it compares with human personality stability.

## Introduction

The turn of the twenty-first century saw an unprecedented interest in non-human animal (hereafter animal) personality. Numerous animal species are now known to display consistent individual variation in behaviour across time and contexts. This individual variation is known to have a wide-ranging impact onanimals, including on measures of fitness and welfare (Dall et al., 2004; Dingemanse & Wolf, 2010; Gosling, 2001; McCowan et al., 2014) and cognition (Lermite et al., 2016).

Understanding animal personality augments our knowledge of the origins of human personality, and comparative studies of personality help us understand development in human personality by providing non-human-centric perspectives (Weiss et al., 2012). Empirical studies examining the comparability of animal and human personality afford insights into the evolutionary trajectory of specific personality traits, as cross-species similarities probably indicate evolutionarily preserved dispositions (Gosling, 2001). Chimpanzees’ phylogenetic proximity to humans makes them a particularly valuable study species in this context, and factor-based instruments similar to those applied to humans have convincingly been applied to chimpanzees. Such studies have established that chimpanzees (and bonobos) display personality differences in traits analogous to the ‘Big Five’, which incorporate agreeableness, conscientiousness, extraversion, neuroticism and openness to experience (Staes et al. 2016; Freeman et al., 2013; King & Figueredo, 1997; Weiss et al., 2012)). Moreover, ratings on these factor-based instruments predict individual differences in great ape cognition (Altschul et al., 2017; Hopper et al., 2014), long-term survival (Altschul et al., 2018) and even brain structure (Latzman et al., 2015), providing further validation of their use.

Despite the recent interest in animal personality, one topic that remains understudied – particularly in great apes – is that of personality stability over substantial time periods. Understanding whether personality remains consistent across the lifespan of great apes allows researchers to document species-specific personality maturation, and to make comparisons with the development and stability of human personality. Cross-sectional studies of great apes reveal that, in chimpanzees, bonobos and gorillas, older individuals are rated as less extraverted than younger individuals (King et al., 2008; Kuhar et al., 2006; Staes et al., 2016; Weiss & King, 2015) – patterns broadly comparable with studies of human personality changes over time (Roberts et al., 2006; Srivastava et al., 2003). Likewise, as with humans, older chimpanzees and bonobos show increased agreeableness (Dutton, 2008; King et al., 2008; Staes et al., 2016; Weiss & King, 2015) and conscientiousness, and decreased neuroticism (King et al., 2008) compared with younger individuals.

Humans and chimpanzees also show some overlap regarding sex differences in age-related variations in personality factors. For instance, in humans (Srivastava et al., 2003; Weisberg et al., 2011) and chimpanzees (King et al., 2008; Weiss & King, 2015), females score higher than males on ratings of agreeableness, and show stronger age-related increases in agreeableness than males. Sex differences in personality are thought to reflect differences in sexual selection (Schmitt et al., 2008) and social factors or life events, such as status competition and cooperation (de Waal, 2000; King et al., 2008; Srivastava et al., 2003), as well as sex differences in human cultural norms and social inequality (Brandt & Henry, 2012; Wood & Eagly, 2002). Hence, while further research is needed, the above data suggest some personality factors reflect evolutionary continuity between humans and chimpanzees (Weiss & King, 2015).

Few studies have taken a longitudinal approach to measure great ape personality, particularly those using factor-based instruments analogous to the human Big Five. In a recent study, 24 chimpanzees from Gombe were rated on the Hominoid Personality Questionnaire (HPQ) – a non-human primate-adapted version of the Big Five, plus dominance. These ratings were compared with ratings taken almost 40 years earlier with the same chimpanzees on the Emotions Profile Index (EPI) (Weiss et al., 2017). Several dimensions were significantly correlated across the two instruments and time periods. For instance, EPI ratings of trustful, aggressive and gregarious were significantly positively correlated with HPQ ratings of agreeableness, neuroticism and extraversion, respectively, while timid and depressed (EPI) were negatively correlated with openness and agreeableness (HPQ), respectively. These correlations suggest convergent validity between different measures and may indicate that some traits, such as aggressiveness and gregariousness, remained stable over time. However, it is difficult to directly assess the stability of personality traits using instruments based on different ratings systems, and this may explain why some expected correlations were not manifest (e.g. a negative correlation between distrustful and agreeableness), and some unexpected correlations appeared (e.g. between gregariousness and agreeableness).

Among captive chimpanzees, Dutton (2008) found that correlations were strong for individual traits over a three-year period for 23 chimpanzees, but for some traits (persistent, adaptable, avoids aggression, moody, socially withdrawn and fearful) stability was comparatively weak. Similarly, King et al. (2008) rated 51 chimpanzees over a mean interval of 6.8 years on an instrument containing the Big Five plus dominance, finding relative stability over the intervals, with some evidence that conscientiousness and extraversion decreased over time. As with Dutton (2008), males exhibited a stronger increase in dominance over the study period, although females showed a stronger increase in agreeableness than males. The mixed findings and methods outlined above from longitudinal research means that drawing firm conclusions, for comparison with cross-sectional data, remains difficult.

When considering behavioural measures of personality (rather than ratings), chimpanzees appear to show stability over short, intermediate and longer time points. For instance, chimpanzees displayed temporal consistencies over two-week (Uher et al., 2008) and three-year (Massen et al., 2013) periods, for various experimentally induced situations (e.g. approaching novel stimuli or foods, reactions to humans, problem solving, tool use behaviours). Similarly, over a six- to eight-year period, individual differences in post-conflict consolation behaviours of captive chimpanzees remained moderately consistent (Webb et al., 2017). Further work is required, however, assessing behavioural stability over longer time points to verify these findings.

Another important reason for establishing personality consistency in animals is to assess the reliability of using previously collected personality data when testing for relationships between personality and other variables. Personality data across a range of animal species has been applied to study topics including disease immunity (Capitanio, 2011; Koolhaas, 2008; Wallis et al., 2018), welfare and conservation (Boissy & Erhard, 2014; Gartner & Weiss, 2018) and sociality (Koski, 2011; Massen & Koski, 2014; Planas-Sitjà et al., 2018; von Merten et al., 2017). Recently, there has been particular focus on examining whether animal personality predicts cognitive performance (for a review, see Dougherty & Guillette, 2018). Great ape studies, using personality data collected (often several) years prior to measurement of the cognitive performance variable, have reported a relationship between personality and participation on cognitive touchscreen tasks (Altschul et al., 2017; Herrelko et al., 2012), response to inequity (Brosnan et al., 2015), puzzle-box interaction success (Hopper et al., 2014) and interaction/success with tools and tool-use tasks (Massen et al., 2013). Although these studies highlight the importance of considering personality when drawing conclusions from cognitive experiments in general (Altschul et al., 2017; Morton et al., 2013), it is apparent that the original personality data may not be representative of the individuals at the time of cognitive investigation.

The present study is a longitudinal assessment of stability of personality in a population of captive chimpanzees. The personality instrument used in the current study measured six personality factors based on the Big Five: agreeableness (being considerate, consoling and protective), dominance (being bold, agonistic and dominant), extraversion (being active, playful, affiliative and sociable), methodical (being goal-orientated and self-caring), openness (being curious, inventive, exploratory and intelligent) and reactivity/undependability (being manipulative, jealous, temperamental and impulsive). These are the same chimpanzees and the same personality instrument that have been examined in previous studies of the relationship between personality and cognitive behaviours (Brosnan et al., 2015; Hopper et al., 2014). Further, the chimpanzees in question are known to exhibit consistent individual differences in social learning behaviours over an overlapping 12-year period (Watson et al., 2018).

The four broad aims of this study were to: (a) provide further longitudinal data to increase knowledge, regarding great ape personality stability over time, particularly assessing factors analogous to the Big Five; (b) grant insights into how factors change over time among males and females, and how this compares with humans; (c) produce richer insights into chimpanzee personality using a variety of methodical approaches to assess long-term stability; and (d) assess the suitability of drawing conclusions informed by personality data collected several years prior to cognitive testing. Based on previous studies of great apes’ personality stability, we considered two main hypotheses. First, we hypothesized that personality traits would show changes over time, predicting that chimpanzees would be rated as more dominant, and less extraverted, on the later assessment than on the first (King et al., 2008; Weiss et al., 2011; Weiss & King, 2015). Second, there would be sex differences in overall ratings and the trajectory of personality traits, predicting that (a) males would be rated as more dominant and more extraverted than females (King et al., 2008; Weiss & King, 2015) and (b) females would be rated as higher in openness and agreeableness than males (Weisberg et al., 2011) and (c) would show an increase in agreeableness over the time period, while males would not (King et al., 2008; Weiss & King, 2015).

## Methods

### Subjects

We studied 50 chimpanzees (25 males) housed in multiple social groups at the National Center for Chimpanzee Care (NCCC), Bastrop, Texas, USA. Most chimpanzees were captive-born and mother-reared and had been housed at the facility for the entire 10 year study period. The chimpanzees’ personality was rated at two separate time points: first (T1), between April 2006 and December 2008 (Freeman et al., 2013) when all participants had been housed at the facility for several years, and second (T2) between September 2015 and December 2016. At the start of T1 (April 2006), chimpanzees ranged from 5.09 to 39.27 years old (mean, *M* = 18.45 years, standard deviation, SD = 7.50), and at the start of T2 (September 2015), the chimpanzees ranged from 14.51 to 50.70 years old (*M* = 28.12 years, SD = 8.04). The breakdown of mean age by sexes is as follows: T1, males *M* = 18.00 (SD = 7.39), females *M* = 18.89 (SD = 7.72); T2, males *M* = 27.42 (SD = 7.39), females *M* = 28.82 (SD = 8.73).

During the approximately 10 year period between T1 and T2, some subjects traversed age categories (see Supplementary Information 1.1). Specifically, at T1, four individuals were classed as juveniles, 20 as adolescents and 26 as adults (in all categories the numbers of males and females were exactly evenly split). At T2, all subjects were classified as adults (i.e. 16 years or older). Further, all subjects experienced changes in group dynamics (either new members added or existing members moved to other groups or deceased, and/or a combination of these). At T1, the sizes of the study groups ranged from three to 14 subjects (*M* = 6.33, SD = 3.00), while at T2, group sizes ranged from eight to 10 subjects (*M* = 8.33, SD = 0.82). At T2, subjects were housed with a mean of 4.48 group members that differed from T1 (SD = 2.06, range = 1–8 different members) and with a mean of 4.55 same group members as T1 (SD = 3.08, range = 0–9 same members). At T1, chimpanzees came from nine groups, and made up an average of 48% of each group (range = 13–90%). AT T2, chimpanzees came from six groups and all members of all groups are included (i.e. the study sample was all members of each of the six groups).

### Materials and procedure

#### Personality instrument

Chimpanzees were rated by human care-staff on a 40-item, seven-point Likert scale questionnaire developed by Freeman et al. (2013). The questionnaire measured six overall traits: agreeableness, dominance, extraversion, methodical, openness and reactivity/undependability. The scale was generated from data collected on the NCCC chimpanzees across a two-stage process between April 2006 and December 2008 (T1). First, a broad corpus of descriptors was produced, based on chimpanzee ethograms, previous research and expert knowledge. Next, to minimize redundancy, three experts selected 41 of the items to comprise the final scale (Table 1). The trait ‘predictable’ was initially included in the instrument but was subsequently removed owing to low reliability, leaving 40 items (Freeman et al., 2013). The six factors obtained though principal component analysis were then validated (at T1) with independently collected behavioural measurements (Freeman et al., 2013). For instance, extraversion was positively correlated with contact aggression, sexual behaviour, begging and play, while dominance was positively correlated with aggressive and displaying behaviours and negatively correlated with submissive behaviours. Agreeableness positively correlated with affiliation and negatively correlated with displace and solicit. Methodical negatively correlated with intervene, reactivity/undependability was positively associated with aggressive behaviours such as display, intervene and sexual behaviour, and was negatively associated with post-conflict affiliation. Finally, openness positively correlated with submissive and playful, and negatively correlated with proximity and social groom (for full details of the behavioural validation process, see Freeman et al. 2013). AT T2, ratings were collated and compared with the ratings collected on the same 40 item instrument approximately 10 years previously. The six factors based on Table 1 were obtained using a process in which only the items that loaded most heavily on a particular factor were counted towards that factor (Hopper et al. 2018; Brosnan et al., 2015; Hopper et al., 2014, 2018; Reamer et al., 2014). For instance, inventive loaded most heavily on to openness, and active loaded most heavily on to extraversion and so on (for all trait-factor loadings from T1, see Supporting Information 2.2).

**Table 1.** The six personality factors with their corresponding traits, based on highest trait loadings from Freeman et al. (2013). (−) denotes negative loadings such that these traits negatively correlated with their factors, e.g. the trait ‘anxious’ negatively correlated with the factor dominance. The trait ‘predictable’ was initially included in the instrument but was subsequently removed from the owing to low reliability (Freeman et al., 2013)

Agreeableness	Dominance	Extraversion	Methodical	Openness	Reactivity/undependability
Considerate	Anxious (−)	Active	Methodical	Affectionate/friendly	Aggressive
Protective	Bold	Affiliative	Self-caring	Human orientated	Autistic
	Cautious (−)	Depressed (−)		Inquisitive/curious	Bullying
	Dependent (−)	Playful		Intelligent	Calm (−)
	Dominant	Sexual		Inventive	Deceptive
	Fearful (−)	Solitary (−)		Persistent	Defiant
	Relaxed				Eccentric
	Timid (−)				Excitable
					Impulsive
					Irritable
					Jealous
					Manipulative
					Mischievous
					Socially inept
					Stingy
					Temperamental/moody

These six factors (agreeableness, dominance, extraversion, methodical, openness and reactivity/undependability) are largely comparable with the human Big Five (agreeableness, conscientiousness, extraversion, openness to experience and neuroticism). In human research, agreeableness captures being kind, considerate and prosocial, extraversion captures being active, social and assertive, openness to experience captures being creative, curious and exploratory while neuroticism captures being emotionally unstable, temperamental and irritable. These human-based factors show strong overlap with the factors agreeableness, extraversion, openness and reactivity/undependability used in this study. In human research, conscientiousness denotes being goal-orientated, organized and planful, which shows some overlap with methodical. Dominance is not typically found on measures of human personality, but captures a combination of extraversion (low caution, bold, assertive) and low neuroticism (low fear and anxiety).

#### Personality ratings

Ratings for T1 and T2 were collected during weekly staff meetings. Raters were either care-staff or supervisory staff, all of whom had worked daily with the chimpanzees for at least six months. At T1, the 17 raters had worked with the chimpanzees for 6 months to 21 years, and rated eight to 10 chimpanzees each week as part of a study investigating personality in a larger number of the NCCC chimpanzees (Freeman et al., 2013). At T2, the eight raters had worked with the chimpanzees for 6 months to 19 years and rated three to five chimpanzees each week. Four raters were present at both T1 and T2, providing some consistency in raters across time points. All raters at T1 and T2 rated all chimpanzees in this study. Raters were instructed to rate chimpanzees based on their overall experience of a chimpanzees’ typical behaviours and interactions, rather than specific and/or recent experiences, and were explicitly instructed not to discuss ratings with each other (see Supporting Information 1.2 for the questionnaire used).

There are two main approaches to measure personality consistency over time. Group-level stability measures the extent to which populations of individuals change over time on personality dimensions. In contrast, rank-order stability reflects the extent to which groups (in this case the entire study population) of individuals maintain similar rank ordering (i.e. ordinal positions) on personality dimensions over time. To assess personality stability at the global and individual-levels, we examined both the mean and individual-level stability.

#### Statistical analysis

We first report the reliability of ratings for T1 and T2 separately, before reporting the mean rank, rank-order stability and individual stability data as measures of consistency over time. For reliability measures, consistent with other studies on non-human primate personality (Freeman et al., 2013), intra-class correlation coefficients (ICCs) are provided to give a measure of inter-rater reliability between chimpanzee care-staff on all factors, where values closer to 1 suggest stronger reliability between raters. To allow comparison with the T1 data, we use two methods, ICC (3,1), which estimates reliability ratings of one individual, and ICC (3,*k*), where reliability is calculated using the average of the *k* raters’ ratings (see Supporting 2.2 for information on how ICC (3,1) and (3,*k*) are each calculated). Following Koo and Li (2016), we interpret ICCs as follows: less than 0.5 as poor reliability, 0.5–0.75 as moderate, 0.75–0.9 as good and greater than 0.9 as excellent reliability.

To compare the stability of the six personality factors across the two time points, overall mean rater scores for each of the six factors (based on the highest trait loadings) were calculated for all chimpanzees (Freeman et al., 2013; Latzman et al., 2015). Specifically, each chimpanzee was given a mean score (ranging from 1 to 7) for each of the six factors, which was the mean score of the respective traits loading on to each of the six factors, as defined by Freeman et al. (2013). To prevent alpha inflation arising from multiple comparisons, we used a false discovery rate control (Storey, 2002), set at 10% (as recommended by McDonald, 2009), which calculates the expected proportion of false positives (rejections of the null hypotheses) from all discoveries. False discovery rate ‘families’ were selected to match their lines of analyses, such that overall mean rank stability reflected a family, as did both assessment of sex differences and rank-order stability analysis.

Group-level stability was assessed by comparing overall mean scores for each of the six traits at T1 and T2 such that if a mean rating of a trait changed from (for example) 4.1 to 4.6, this would represent an increase of 0.5 on the scale. Mixed effects ANOVAs were conducted: the two time points were the within-subjects independent variable, sex was the between-subjects independent variable and personality rating was the dependent variable. We first report the main effects of whether each of the six personality factors remained stable and then, for each factor, sex differences are examined by analysing both overall main effects of sex and sex by time interactions. We finish by reporting stability of personality for males and females separately.

To assess rank-order stability, we examined intra-class correlations between individuals across the two rating periods (Dingemanse & Dochtermann, 2013; Dutton, 2008; King et al., 2008; Koski, 2011; Uher, 2013). To account for variance in ratings owing to different raters rating subjects at T1 and T2, we calculated ICCs (3,*k*) for all raters combined (*N* = 50 chimpanzees), for those chimpanzees who were rated by the same raters at both time points (*N* = 14) and for chimpanzees (*N* = 36) whose raters differed at T1 and T2 (King et al., 2008).

For further analysis of individual-level stability, we also calculated the reliable change index (RCI) (Jacobson & Truax, 1991). The RCI is used to distinguish individual change that is statistically significant from change that may have occurred owing to measurement error. For each individual subject, the difference in ratings from T2 to T1 was compared with the distribution of change scores expected solely by measurement error (RCI = (T2 score − T1 score)/standard error of the measurement of the difference; see Supporting Information 2.1 for further information on the RCI calculation). Using a 95% confidence interval, for each factor individuals were classified as having ‘increased’, ‘decreased’, or stayed the ‘same’ on each factor (Pullmann et al., 2006).

## Results

### Reliability of ratings

For T1 (Freeman et al., 2013), the ICCs (3,1) and (3,*k*) were as follows: agreeableness (0.37, 0.51), dominance (0.48, 0.64), extraversion (0.48, 0.65), methodical (0.28, 0.36), Openness (0.49, 0.63) and reactivity/undependability (0.48, 0.61), For T2, the ICC (3,1) and (3,*k*) were as follows: agreeableness (0.57, 0.72), dominance (0.43, 0.84), extraversion (0.24, 0.61), methodical (0.25, 0.41), openness (0.43, 0.79) and reactivity/undependability (0.37, 0.90). See Supporting Information 2.2 for the intra-class correlation coefficients values (3,1) and (3,*k*) for all individual traits at T1 and T2.

### Mean-rating consistency

#### Main effects over time

Table 2 provides a breakdown of the overall mean scores for the six factors at T1 and T2. Mean scores of agreeableness (*F*_1,48_ = 6.33 *p* = 0.015) and reactivity/undependability (*F*_1,48_ = 54.08, *p* < 0.001) decreased significantly overall from T1 to T2. There was also a significant increase in mean scores of dominance (*F*_1,48_ = 43.83, *p* < 0.001) from T1 to T2, whereas extraversion, methodical and openness did not differ between T1 and T2.

**Table 2.** Mean scores (SD) of each of the six factors at T1 (April 2006 to December 2008) and T2 (September 2015-December 2016), overall and for males and females. Mean-order stability demonstrates the group-level T1 and T2 scores (on a scale of 1–7) and change over the 10 year time point for each factor. Significant differences between T1 and T2 indicated as *p < 0.05, **p < 0.01 and ***p < 0.001

	Overall mean scores			Males			Females		
Factor	T1	T2	T1 − T2	T1	T2	T1 − T2	T1	T2	T1 − T2
Agreeableness	4.32 (0.46)	4.11 (0.62)	−0.21*	4.25 (0.44)	3.78 (0.53)	−0.48***	4.39 (0.48)	4.44 (0.65)	+0.05
Dominance	4.19 (0.59)	4.64 (0.47)	+0.45***	4.46 (0.53)	4.99 (0.51)	+0.53***	3.91 (0.53)	4.29 (0.80)	+0.38***
Extraversion	4.78 (0.47)	4.69 (0.44)	−0.08	4.93 (0.42)	4.85 (0.42)	−0.08	4.61 (0.47)	4.53 (0.40)	−0.08
Methodical	4.65 (0.38)	4.51 (0.67)	−0.14	4.63 (0.44)	4.54 (0.43)	−0.09	4.66 (0.33)	4.70 (0.42)	+0.04
Openness	4.73 (0.53)	4.75 (0.62)	+0.02	4.81 (0.52)	4.65 (0.65)	−0.16	4.64 (0.53)	4.84 (0.60)	+0.20
Reactivity/undependability	3.90 (0.47)	3.33 (0.59)	−0.57***	4.01 (0.43)	3.48 (0.57)	−0.53***	3.80 (0.51)	3.18 (0.60)	−0.62***

#### Sex differences

Table 2 provides a breakdown of the overall mean scores for the six factors at T1 and T2 for males and females. To examine sex differences in personality, we looked at main effects of sex, time by sex interactions and where appropriate, within-sex effects for each factor.

##### Agreeableness

**Figure 1.**
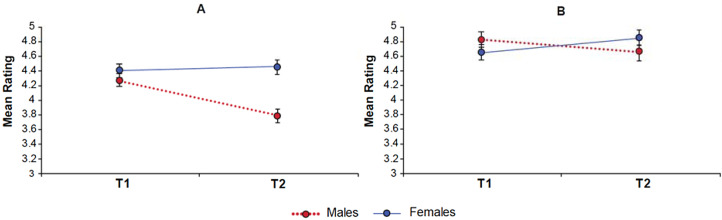
Results revealed significant sex by time interactions for agreeableness (a) and openness (b)

Males were rated as significantly less agreeable than females across T1 and T2 combined (*F*_1,48_, = 10.63, *p* = 0.002). There was also a significant interaction between time and sex (Figure 1), such that males exhibited a decrease of 0.48 and females displayed a slight increase of 0.05 (*F*_1,48_ = 9.77, *p* = 0.003). The decrease in male agreeableness from T1 to T2 was significant (*F*_1,24_ = 20.41, *p* < 0.001) but the increase in females was not.

##### Dominance

Males were rated as more dominant than females across T1 and T2 combined (*F*_1,48_ = 9.74, *p* < 0.001). There was no significant interaction between time and sex but both male and female ratings of dominance increased significantly (males, F_1,24_ = 57.23, *p* < 0.001; females, F_1,24_ = 10.12, *p* = 0.004).

##### Extraversion

Males were rated as more extraverted than females across T1 and T2 combined (*F*_1,48_ = 9.53, *p* = 0.003). There was no sex by time interaction, nor did male or female ratings differ between T1 and T2.

##### Openness

There was no main effect of sex but there was a sex by time interaction (*F*_1,48_ = 4.67, *p* = 0.036) such that males exhibited a decrease of 0.16 and females an increase of 0.20 from T1 to T2. The decrease in male openness only approached significance (*F*_1,24_ = 4.02, *p* = 0.056), while the increase in females was not significant.

##### Reactivity/undependability

There was no main effect of sex or a sex by time interaction. However, ratings decreased significantly from T1 to T2 for both sexes (males, *F*_1,24_ = 24.46, *p* < 0.001; females, *F*_1,24_ = 32.14, *p* < 0.001).

##### Methodical

There were no significant effects.

To assess whether individuals changed more within or between age category, we conducted additional analysis looking at time by age category interactions. Although small sample sizes preclude making firm conclusions, no time by age category interactions were significant (all *p* values >0.05; see Supporting Information Table S4 for means for T1 and T2, and for T2 − T1 by age category for each factor).

### Rank-order stability

Table 3 presents the rank-order stability results. When all raters were combined, dominance (ICC 3, *k* = 0.854) showed the highest (good) intra-class correlation coefficient between T1 and T2, and methodical (ICC 3, *k* = 0.493), showed the lowest (poor) rank-order stability. The other four factors all showed moderate rank-order stability (ICC 3, *k* range = 0.535–0.712), suggesting individuals were variable in their rank-order position over time. For four of six factors, ICCs were stronger when analysis was restricted to raters who were present at both time points (ICC 3, *k* range = 0.479–0.631) compared with the case where raters differed (ICC 3, *k* range = 0.025–0.824).

**Table 3.** Overview of results from individual analyses. For rank-order stability, ‘All’ represents intra-class correlation coefficients (ICC) correlations for all raters combined (*N* = 50 chimpanzees), ‘same’ represents chimpanzees who were rated by the same raters at both time points (*N* = 14), and ‘different’ represents chimpanzees whose raters differed at T1 and T2 (*N* = 36). The reliable change index (RCI) provides the percentage of individuals that significantly increased, stayed the same or decreased over the study period according to the RCI calculation

	Rank order stability (raters)			RCI (%)		
Factor	All (*N* = 50)	Same (*N* = 14)	Different (*N* = 36)	Increased	Same	Decreased
Agreeableness	0.535	0.551	0.309	2	88	10
Dominance	0.854	0.529	0.551	18	82	0
Extraversion	0.712	0.479	0.589	2	98	0
Methodical	0.493	0.515	0.025	4	94	2
Openness	0.596	0.493	0.824	10	82	8
Reactivity/undependability	0.661	0.631	0.559	2	42	56

### RCI

**Figure 2.**
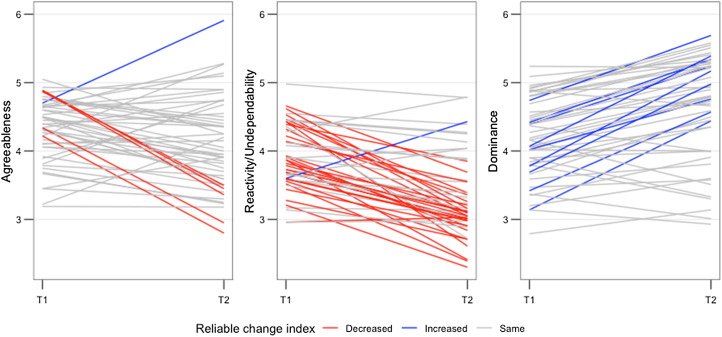
Individual reliable change index (RCI) values for agreeableness, dominance and reactivity/undependability, which all showed significant mean level change over time. Red lines show individuals whose RCI value significantly decreased, blue lines indicate individuals whose RCI value significantly increased and grey lines indicate individual's whose RCI value did not change significantly over the time points

The extent to which individuals change (T1 to T2) was over the RCI threshold varied by factor. Reactivity/undependability was the factor for which most individuals changed, with 58% passing the RCI threshold in either direction. Dominance (18%), openness (18%) and agreeableness (12%) showed lower individual-level change, and methodical (6%) and extraversion (2%) showed the lowest rates of individual change over time. Thus, while reactivity/undependability, agreeableness and dominance all showed overall (group) mean level change from T1 to T2, only for reactivity/undependability did the majority (and by a small margin) of individuals show significant change according to the RCI (Figure 2). Table 3 presents the group RCI scores and Table 4 presents RCI group by the sexes, and for a full breakdown of RCI scores by age category see Supporting Information Table S5.

**Table 4.** Breakdown of reliable change index scores by sexes

	RCI (%)					
	Males			Females		
Factor	Increased	Same	Decreased	Increased	Same	Decreased
Agreeableness	2	90	8	0	98	2
Dominance	10	90	0	8	92	0
Extraversion	2	98	0	0	100	0
Methodical	0	98	2	4	96	0
Openness	2	94	4	8	88	4
Reactivity/Undependability	2	70	28	0	72	28

## Discussion

### Stability of chimpanzees’ personality over time; overall and sex differences

We examined the stability of multiple chimpanzee personality traits by measuring changes in factors across an approximately 10 year period using the same instrument, revealing consistencies and differences with previous work. Analysis of mean rank stability revealed that consistent with previous findings and with our prediction, overall, chimpanzees showed increased dominance with age. Approximately half of the study subjects traversed age categories during the study period (predominantly moving from adolescence into adulthood). Our findings largely fit cross-sectional data on personality development showing that adult chimpanzees are more dominant than juvenile and younger chimpanzees (King et al., 2008) – although we note that no age category by time interactions were found for any of the six factors. Contrary to our prediction, chimpanzees did not show an overall significant decrease in extraversion over time. The chimpanzees were also rated as significantly less reactive/undependable over time – a finding that was also not predicted.

Analyses of sex differences in personality traits also indicated that males and females differed for agreeableness, openness, dominance and extraversion. In line with our predictions, males were rated as more dominant and extraverted than females, and females showed an increase in agreeableness and openness while males did not. The finding that males actually decreased in these factors was not, however, predicted. Further, in contrast to other cross-sectional findings, the chimpanzees were rated as less agreeable over time – although this decrease was driven by males.

### Comparisons with sex differences in chimpanzee and human personality stability

The results revealed some sex differences in personality traits that contrast with previous chimpanzee studies but correspond with findings in humans. For instance, King et al. (2008) found that chimpanzees decline in openness with age, whereas in the present study, while males significantly declined in openness, females increased by a similar margin. Although these findings contrast with those of King and colleagues, they are consistent with findings that human females score higher on openness to experience than males (Weisberg et al., 2011) and that this pattern continues throughout development (Gjerde & Cardilla, 2009). In humans, females score particularly high on the facets of warmth, openness to feelings and aesthetics (Chapman et al., 2007; Costa et al., 2001). It is important to note, however, that vast majority of longitudinal studies of personality in humans are based on Western populations, for whom our human comparisons are based on (and thus limited to), and further research is needed to measure cultural differences in personality stability over time (see Costa et al., 2019 and Supporting Information 3 for additional discussion on variance and invariance of personality across age groups and cultures).

One potential explanation for the contrasting findings between this study and others is that the present study, unlike King et al.'s, included affectionate/friendly as a facet of openness, and thus could contribute to the sex differences found here. Similarly, intelligent and persistent loaded on to openness for our instrument, while both loaded on to dominance in King et al.'s study (for a breakdown of the traits used in both studies, see Supporting Information Tables S6 and S7). However, despite these differences there is also large overlap between the two instruments. For instance, there is large similarity in the traits loading onto the factors agreeableness (protective, kind), dominance (non-fearful and non-submissive, dominant), extraversion (sociable, affiliative, playful, non-depressed) and openness (inventive, inquisitive). Reactivity/undependability also showed overlap with King et al.'s consciousnesses (irritable, jealous, impulsive).

Similarly, it is important to consider differences in age categories, group composition and environmental factors when comparing these data with those of other studies, particularly inbreeding populations. Here, all study subjects experienced changes in group members and group sizes across the study period, and many experienced relocations to new enclosures (on-site). Personality has been shown to correlate with individual differences in stress response in young chimpanzees (Anestis et al., 2006) and it has been found that non-human primate social dynamics including individual and group level affiliative and aggressive behaviours are disrupted by enclosure relocation and changes to group demographics (Dufour et al., 2011; Schel et al., 2013), but that such behaviours and group dynamics begin to return to pre-disruption levels within a year (Schel et al., 2013; Yamanashi et al., 2016). Given there were no major alterations to group demographics or relocations for the study subjects for several years prior to the second data collection period, it is not clear whether the effects of relocation had a major bearing on ratings.

In turn, these findings can contribute to the development of a theoretical framework in which to empirically examine specific hypotheses about chimpanzee personality over time, particularly with regards to ecological and life history changes. For example, future research could examine how individuals high or low in social-based traits such as dominance, agreeableness and extraversion are shaped by adjustments to group dynamics. Tools such as social network analysis have proven useful for helping facilitate and monitor the integration of different groups or relocation of non-human primates (Dufour et al., 2011; Schel et al., 2013) and chimpanzees display ‘friendships’ based on personality homophily (Massen & Koski, 2014). Thus personality instruments may be an important tool for group formations or relocations (Schapiro, 2017). Further, given that these data indicated that reactivity/undependability showed high levels of mean decreases, it may be that individuals high in this trait exhibit lower stability over time than those scoring low in it. These questions would be well suited to longitudinal personality data over multiple time points. Such data, coupled with documentation of major events, including changes to social environments, would allow these types of assessments, and in turn comparisons with analogous human data (Ying & Han, 2006).

Likewise, evolutionary theory suggests that if changes in personality over time are an evolutionary preserved feature of chimpanzees there should be corresponding fitness benefits (Blaszczyk, 2020). While extraversion itself has been linked with longer survival in wild gorillas (Weiss et al., 2013), there has been a striking lack of empirical research assessing fitness benefits of non-human animal personality instability (Blaszczyk, 2020; Trillmich et al., 2018). It is possible, for examples that females – who are the socially dispersing sex in chimpanzees – become more agreeable over adulthood to maximize social bonds. It is important, for evolutionary models of personality, for researchers to document the association between changes in non-human animal personality over time with fitness benefits so such hypotheses can be tested.

Our findings also afford comparisons with other ape species and humans. Assessments of bonobo personality has shown both overlap and differences with human and chimpanzee personality data. For example, while there are similarities in the factors found in bonobos, there are contrasting patterns of sex differences to chimpanzees and humans. Female bonobos score higher on traits such as assertiveness and extraversion than male bonobos and receive less aggression (Staes et al., 2016). Higher female assertiveness and extraversion reflect the fact that, unlike chimpanzees, they are more socially dominant and maintain close relationships with other group members compared with male bonobos (Staes et al., 2016; Vervaecke et al., 2000). Similarly, as with humans, orangutans – for whom factor-based personality traits have also been validated – show age-related declines in extraversion and neuroticism. Male orangutans, like chimpanzees, also score higher in dominance than females (Weiss & King, 2015). Comparisons across different ape species are crucial for understanding evolutionary continuity of personality (Weiss & King, 2015).

### Individual-level change over time: multiple approaches to assessing long-term stability

Investigation of rank-order stability revealed comparatively strong stability for ratings of dominance; individuals who were rated as scoring highly in the factor dominance at T1 were also rated as scoring highly in the factor dominance 10 years later. This finding is perhaps expected: dominance exhibited the strongest rank-order stability in other studies (e.g. King et al., 2008). Extraversion also exhibited relatively high rank-order stability compared with the other traits, also suggesting that individuals high (or low) remained high (or low) in this factor. The other four traits overall exhibited lower rank-order stability, indicating that individuals were variable in their ordinal rank-position consistency when compared at T1 and T2. That methodical displayed the least rank-order consistency (regardless of whether the raters were the same, different or combined) is not surprising. The initial study by Freeman et al. (2013) showed methodical to have the lowest reliability and it failed to correlate with factors from other instruments measuring chimpanzee personality (and thus caution should be exercised when interpreting from this factor, as noted by Freeman and colleagues in the initial study).

When assessing individual-level change using the RCI, despite overall mean changes in dominance, agreeableness and reactivity/undependability, only in the latter trait did most individuals exhibit a change that was considered ‘reliable’. For dominance and agreeableness under 20% of individuals exhibited a statistically significant change over time. This may be because reactivity/undependability included traits such as being excitable, impulsive, aggressive, mischievous, eccentric and calm (negatively loaded) – all traits that perhaps change to a greater extent as subjects traverse age categories than those within dominance and agreeableness.

An understanding of individual-level changes occurring over time compliments our understanding of population changes. Population-level changes of personality may either be driven by a subset of individuals or represent a general group-level trend in change over time (or a combination of both). Discrepancies between population-level and individual-level changes over time have important implications for future research and the conclusions that can be drawn from longitudinal assessments of personality. First, researchers should be cautious when drawing conclusions about population-level changes in personality over time. Although data may indicate that personality may significantly change over time at the population level, this may be driven by certain individuals. Second, presenting individual and population data on all subjects is important to provide a complete picture of the data and how personality changes over time – an approach taken in very few studies. Third, in line with studies with other non-human animals, these findings may indicate that key individuals, in terms of personality scores, may have a significant impact on group behaviours (Aplin et al., 2013; Brown & Irving, 2014; Farine et al., 2015).

In addition to providing insights regarding how group- and individual-level changes in personality interact, our findings build on the existing, yet limited, longitudinal data using factor-based instruments to assess chimpanzee personality. For instance, despite increasing the time scale compared with King et al. (2008) (6.8 vs. 10 years here), when all raters were combined, most of the correlation coefficients were similar to those obtained in their study: 0.85 vs. 0.74 for dominance; 0.66 vs. 0.51 for reactivity/undependability vs. dependability/conscientiousness; 0.60 vs. 0.70 for openness; 0.54 vs. 0.39 for agreeableness; and 0.71 vs. 0.48 for extraversion. Further, as with King et al. (2008) at least half of the traits studied exhibited higher correlation coefficients for data from raters who were present at both time points compared with data from raters who differed. Such closely matched coefficients and findings are indicative of robust validity in findings across measures and chimpanzee populations.

### Drawing conclusions based on personality data collected years prior to cognitive testing

These findings also have implications for the use of personality ratings obtained prior to other types of empirical tests (e.g. cognitive assessments). For example, much recent work has highlighted the importance of openness in chimpanzee problem solving, study participation and success (Altschul et al., 2017; Herrelko et al., 2012; Hopper et al., 2014), and performance on inequity tasks (Brosnan et al., 2015). These studies relied on the personality ratings collected several years prior to the cognitive testing sessions, and indeed, two of these studies used the same subjects and same personality instrument as this study (Brosnan et al., 2015; Hopper et al., 2014). Here, we found that males significantly decreased in openness over several years, while female ratings increased by a similar (although non-significant) margin. This may suggest, depending on the timeframe between rating collection and experimental testing, that the personality ratings may not always accurately reflect the individuals at the time of study participation. Although rating data requires much effort and valuable time from care-staff, we encourage, where possible, (a) authors to use or collect recent personality data when conducting personality-based assessments of cognitive performance or other empirical measurements, and (b) researchers to consider temporal instability in personality measures when drawing conclusions regarding the predictive power of personality for cognitive measures.

Our data revealed important insights regarding stability in chimpanzee personality over an approximately 10 year period. We found group-level changes in three of six personality factors measured (an increase in dominance and decreases in agreeableness and reactivity/undependability), overall sex differences found for three traits (males rated higher than females in dominance and extraversion but lower in agreeableness), and males and females showing different trajectories for two further traits (males decreasing and females increasing in agreeableness and openness) over the 10 year period. Given that several personality factors showed group level changes *and* variable individual stability over time, we suggest that researchers measuring the relationship between personality and cognitive performance in non-human primates obtain the most current personality data possible. The reported sex differences converge with studies of Western humans, providing new longitudinal evidence for an evolutionary basis for the human pattern of age-related fluctuations in male and female personality traits. In turn, these findings lay the foundation of an exciting suite of questions about how environmental and social changes influence chimpanzees with specific personality profiles, and how this compares with data on human personality and environmental and social changes.

## Data Availability

Data has been uploaded to Dryad Repository (doi:10.5061/dryad.xksn02vc0).

## Supplementary material

For supplementary material accompanying this paper visit http://dx.doi.org/10.1017/ehs.2020.45. click here to view supplementary material
