# Supplementary material for: Sex differences in longitudinal personality stability in chimpanzees
Source: Evol Hum Sci. 2020 Sep 9;2:e46. doi: 10.1017/ehs.2020.45 (PMC10427468; doi:10.1017/ehs.2020.45)
Supplement: Supplementary file 1 [file S2513843X20000456sup001.docx]

## Supplementary Material

*SI 1: Supplementary Methods information*

**Supplementary Table 1: The number of subjects by sex and age categories.**

|  | **T1** | | **T2** | |
| --- | --- | --- | --- | --- |
|  | **Males** | **Females** | **Males** | **Females** |
| **Infant (0-4 years)** | 0 | 0 | 0 | 0 |
| **Juvenile (5-8 years)** | 2 | 2 | 0 | 0 |
| **Adolescents (9-15 years)** | 10 | 10 | 0 | 0 |
| **Adults (16+ years)** | 13 | 13 | 25 | 25 |

## SI 1.2: An example of the personality questionnaire administered to carestaff.

**ASSESSMENT OF PERSONALITY IN CHIMPANZEES**

Chimpanzee personality assessments can be made with this questionnaire by assigning a numerical score for all of the personality traits listed on the following pages. Make your judgments on the basis of your own understanding of the trait guided by the short clarifying definition following each trait. The chimpanzee’s own behaviors and interactions with other chimpanzees should be the basis for your numerical ratings. Use your own subjective judgment of typical chimpanzee behavior to decide if the chimpanzee you are scoring is above, below, or average for a trait. The following seven-point scale should be used to make your ratings.

1. **Displays either total absence or negligible amounts of the trait.**
2. **Displays small amounts of the trait on infrequent occasions.**
3. **Displays somewhat less than average amounts of the trait.**
4. **Displays about average amounts of the trait.**
5. **Displays somewhat greater than average amounts of the trait.**
6. **Displays considerable amounts of the trait on frequent occasions.**
7. **Displays extremely large amount of the trait.**

Please give a rating for each trait even if your judgment seems to be based on a purely subjective impression of the chimpanzee and you are somewhat unsure about it. Indicate your rating by placing a cross in the box underneath the chosen number.

Finally, do not discuss your rating of any particular chimpanzee with anyone else. This restriction is necessary in order to obtain valid reliability coefficients for the traits.

Chimpanzee’s name _________________________ Rater’s name__________________

Date___________

**Active**: Spends little time idle and seems motivated to spend considerable time either moving around or engaging in some overt, energetic behavior.

**1 2 3 4 5 6 7**

Least Most

________________________________________________________________________

**Affectionate/Friendly**: Seems to have a warm attachment or closeness with other chimpanzees. This may entail frequent grooming, touching, embracing, or lying next to others.

**1 2 3 4 5 6 7**

Least Most

________________________________________________________________________

**Affiliative**: Agreeable, sociable. Appears to like the company of others. Seeks out social contact with, or showing preference for, another animal; for example, playing walking next to, or sitting with another animal.

**1 2 3 4 5 6 7**

Least Most

________________________________________________________________________

**Aggressive**: Often initiates fights or other menacing and agonistic encounters with other chimpanzees

**1 2 3 4 5 6 7**

Least Most

________________________________________________________________________

**Anxious**: Hesitant, indecisive, tentative, jittery.

**1 2 3 4 5 6 7**

Least Most

________________________________________________________________________

**Autistic**: Does not make eye contact, and/or not well integrated into social group.

**1 2 3 4 5 6 7**

Least Most

________________________________________________________________________

**Bold**: Daring, not restrained or tentative. Not timid, shy or coy.

**1 2 3 4 5 6 7**

Least Most

**________________________________________________________________________**

**Bullying**: Overbearing and intimidating towards younger or lower ranking chimpanzees.

**1 2 3 4 5 6 7**

Least Most

________________________________________________________________________

**Calm**: Equable, restful: Reacts to others in an even, calm way; is not easily disturbed or agitated.

**1 2 3 4 5 6 7**

Least Most

________________________________________________________________________

**Cautious**: Exhibits a more careful measured approach to investigating things.

**1 2 3 4 5 6 7**

Least Most

________________________________________________________________________

**Considerate/Kind**: Often consoles others in distress to provide reassurance.

**1 2 3 4 5 6 7**

Least Most

________________________________________________________________________

**Deceptive**: Deceives others for his/her own benefit.

**1 2 3 4 5 6 7**

Least Most

________________________________________________________________________

**Defiant**: Assertive or contentious in a way inconsistent with the usual dominance order. Maintains these actions despite unfavorable consequences or threats from others.

**1 2 3 4 5 6 7**

Least Most

________________________________________________________________________

**Dependent**: Often relies on other chimpanzees for leadership, reassurance, touching, embracing and other forms of social support.

**1 2 3 4 5 6 7**

Least Most

________________________________________________________________________

**Depressed**: Often appears isolated, withdrawn, sullen, brooding and has reduced activity.

**1 2 3 4 5 6 7**

Least Most

________________________________________________________________________

**Dominant**: Able to displace, threaten or take food from other chimpanzees. Or subject may express high status by decisively intervening in social interactions.

**1 2 3 4 5 6 7**

Least Most

________________________________________________________________________

**Eccentric**: Shows stereotypies or unusual mannerisms.

**1 2 3 4 5 6 7**

Least Most

________________________________________________________________________

**Excitable**: Easily aroused to an emotional state.

**1 2 3 4 5 6 7**

Least Most

________________________________________________________________________

**Fearful**: Subject reacts excessively to real or imagined threats by displaying behaviors such as screaming, grimacing, running away or other signs of anxiety or distress.

**1 2 3 4 5 6 7**

Least Most

_______________________________________________________________________

**Human oriented**: Very interested in human activities around their enclosure. Solicits support from humans.

**1 2 3 4 5 6 7**

Least Most

**Impulsive**: Often displays some spontaneous or sudden behavior that could not have been anticipated.

**1 2 3 4 5 6 7**

Least Most

________________________________________________________________________

**Inquisitive/Curious**: Readily explores new situations, objects or animals.

**1 2 3 4 5 6 7**

Least Most

________________________________________________________________________

**Intelligent**: Quick and accurate in judging and comprehending both social and nonsocial situations.

**1 2 3 4 5 6 7**

Least Most

________________________________________________________________________

**Inventive**: More likely than others to engage in novel behaviors. E.g. Using new devices or materials in their enclosure.

**1 2 3 4 5 6 7**

Least Most

________________________________________________________________________

**Irritable**: Often seems in a bad mood or is impatient and easily provoked to anger exasperation and consequent agnostic behavior.

**1 2 3 4 5 6 7**

Least Most

________________________________________________________________________

**Jealous/Attention-seeking**: Often troubled by others who are in a desirable or advantageous situation such as having food, a choice location or access to social groups. May attempt to disrupt activities or make noise to get attention.

**1 2 3 4 5 6 7**

Least Most

________________________________________________________________________

**Manipulative**: Is able to get others to do things without using force.

**1 2 3 4 5 6 7**

Least Most

________________________________________________________________________

**Methodical**: Does things in a logical, organized manner following a consistent goal.

**1 2 3 4 5 6 7**

Least Most

________________________________________________________________________

**Mischievous**: Engages in activities or behavior with the goal of provoking negative reactions from someone or doing something that has previously been established as not socially acceptable.

**1 2 3 4 5 6 7**

Least Most

________________________________________________________________________

**Persistent**: Tends to continue in a course of action, task, or strategy for a long time or continues despite external interference.

**1 2 3 4 5 6 7**

Least Most

________________________________________________________________________

**Playful**: Is eager to engage in lively, vigorous, sportive or acrobatic behaviors with or without other chimpanzees.

**1 2 3 4 5 6 7**

Least Most

________________________________________________________________________

**Predictable**: Behavior is consistent and steady over extended periods of time. Does little that is unexpected or deviates from its usual behavioral routine.

**1 2 3 4 5 6 7**

Least Most

________________________________________________________________________

**Protective**: Shows concern for other chimpanzees and often intervenes to prevent harm or annoyance from coming to them.

**1 2 3 4 5 6 7**

Least Most

________________________________________________________________________

**Relaxed**: Does not show restraint in postures and movements. Is not tense.

**1 2 3 4 5 6 7**

Least Most

________________________________________________________________________

**Self-caring**: Shows high, but healthy level of self-grooming and cleanliness.

**1 2 3 4 5 6 7**

Least Most

**Sexual**: Engages in frequent copulations and/or masturbation.

**1 2 3 4 5 6 7**

Least Most

________________________________________________________________________

**Socially-inept**: Acts inappropriately in a social setting.

**1 2 3 4 5 6 7**

Least Most

________________________________________________________________________

**Solitary**: Prefers to spend considerable time alone not seeking or avoiding contact with other chimpanzees.

**1 2 3 4 5 6 7**

Least Most

________________________________________________________________________

**Stingy**: Is excessively desirous or covetous of food, favored locations, or other resources in enclosure. Is unwilling to share these resources with others.

**1 2 3 4 5 6 7**

Least Most

**________________________________________________________________________**

**Temperamental**/**Moody**: Is inconsistent and wildly/varying in its moods and behaviors.

**1 2 3 4 5 6 7**

Least Most

________________________________________________________________________

**Timid**: Lacks confidence is easily alarmed and is hesitant to venture into new social or nonsocial situations.

**1 2 3 4 5 6 7**

Least Most

________________________________________________________________________

## SI 2: Supplementary results information

**SI 2.1 Information about the reliable change index (RCI).**

An RCI indicates whether a change in an individual’s score is statistically significantly greater than a difference due to random measurement error alone.

The Reliable Change Index is calculated as follows:

x(T2) −x (T1)

___________________________________

Standard error of measurement of the difference

Where x(T1) and x(T2) are measurement scores for an individual at different points in time.

## SI 2.2 Information on intra-class correlation coefficients and personality ratings.

We use two methods, ICC (3,1), where subjects are assessed by each rater, and the raters are the only raters of interest, and ICC (3,k), where reliability is calculated using the average of the k raters’ ratings. ICC (3,1) and (3,k) are calculated as follows (Koo & Li, 2016):

3,1 = MS_R_-MS_E_ / MS_R_+(*k*-1)MS_E_

3,k = MS_R_-MS_E_ / MS_R_

Where MS_R_ refers to the mean square for rows, MS_E_ refers to the mean square for error and k refers to number of raters/measurements.

## Supplementary Table 2: ICC (3,k) inter-rater reliability values for T1 between April 2006-December 2008 (from Freeman et al. 2013) and for T2, between September 2015-December 2016.

| **Personality trait** | **ICC (3,k) values from T1:**  **2006-2008** | **ICC (3,1) values from T1:**  **2006-2008** | **ICC (3,k) values from**  **T2:**  **2015-2016** | **ICC (3,1) values from**  **T2:**  **2015-2016** |
| --- | --- | --- | --- | --- |
| Active | .85 | .26 | .84 | .35 |
| Affectionate/Friendly | .55 | .07 | .68 | .17 |
| Affiliative | .42 | .04 | .63 | .14 |
| Aggressive | .67 | .11 | .91 | .50 |
| Anxious | .60 | .09 | .85 | .36 |
| Autistic | .35 | .03 | .35 | .05 |
| Bold | .80 | .20 | .87 | .41 |
| Bullying | .76 | .16 | .91 | .50 |
| Calm | .56 | .07 | .78 | .26 |
| Cautious | .70 | .13 | .82 | .31 |
| Considerate/Kind | .48 | .06 | .79 | .27 |
| Deceptive | .58 | .08 | .96 | .69 |
| Defiant | .55 | .07 | .89 | .44 |
| Dependent | .56 | .07 | .78 | .28 |
| Depressed | .52 | .06 | .41 | .07 |
| Dominant | .85 | .26 | .89 | .45 |
| Eccentric | .44 | .05 | .70 | .19 |
| Excitable | .68 | .12 | .67 | .17 |
| Fearful | .65 | .11 | .83 | .40 |
| Human oriented | .75 | .16 | .78 | .26 |
| Impulsive | .61 | .09 | .77 | .25 |
| Inquisitive/Curious | .67 | .11 | .63 | .15 |
| Intelligent | .64 | .10 | .79 | .27 |
| Inventive | .66 | .12 | .75 | .23 |
| Irritable | .61 | .09 | .82 | .32 |
| Jealous/Attention-Seeking | .72 | .14 | .82 | .31 |
| Manipulative | .67 | .11 | .82 | .32 |
| Methodical | .36 | .03 | .56 | .11 |
| Mischievous | .71 | .13 | .84 | .35 |
| Persistent | .50 | .06 | .50 | .18 |
| Playful | .71 | .14 | .72 | .21 |
| Protective | .55 | .07 | .72 | .21 |
| Relaxed | .61 | .09 | .73 | .21 |
| Self-caring | .36 | .03 | .56 | .13 |
| Sexual | .66 | .11 | .69 | .18 |
| Socially-inept | .47 | .05 | .39 | .06 |
| Solitary | .60 | .08 | .52 | .10 |
| Stingy | .71 | .14 | .76 | .24 |
| Temperamental/Moody | .65 | .12 | .82 | .31 |
| Timid | .70 | .11 | .87 | .87 |

**Supplementary Table 3: Factor loadings from Freeman et al. (2013)**

Trait Reactivity/ Dominance Extraversion Openness Agreeableness Methodical

Undependability

| Temperamental | **.85** | -.01 | .04 | -.02 | -.08 | .18 |
| --- | --- | --- | --- | --- | --- | --- |
| Irritable | **.87** | -.09 | -.05 | -.09 | -.13 | .15 |
| Deceptive | **.79** | -.13 | .12 | .18 | .18 | .05 |
| Stingy | **.68** | **-.49** | .07 | -.06 | .23 | .03 |
| Manipulative | **.68** | -.35 | .14 | .18 | .25 | .09 |
| Defiant | **.74** | -.06 | .31 | .22 | -.03 | -.10 |
| Jealous | **.70** | -.07 | .35 | **.40** | .02 | -.05 |
| Bullying | **.68** | **-.56** | .25 | -.07 | .06 | .03 |
| Aggressive | **.66** | **-.42** | **.41** | -.12 | -.06 | .14 |
| Impulsive | **.77** | -.04 | .28 | .27 | .00 | -.12 |
| Socially Inept | **.58** | .36 | .02 | .07 | -.37 | -.06 |
| Mischievous | **.73** | .07 | **.41** | .33 | -.01 | -.13 |
| Eccentric | **.62** | .13 | -.22 | .36 | -.04 | -.14 |
| Dependent | -.02 | **-.76** | .21 | .01 | .30 | -.15 |
| Fearful | .03 | **-.88** | -.11 | .05 | -.12 | -.01 |
| Timid | -.14 | **-.84** | -.27 | -.23 | -.09 | -.04 |
| Cautious | -.23 | **-.81** | -.11 | -.13 | .07 | -.01 |
| Anxious | .32 | **-.75** | .28 | -.07 | -.05 | .20 |
| Dominant | **.40** | **.78** | .16 | -.03 | .18 | .13 |
| Bold | **.53** | **.61** | .35 | .29 | .12 | .05 |

| Human oriented | .08 | -.14 | -.02 | **.83** | .01 | .05 |
| --- | --- | --- | --- | --- | --- | --- |
| Inquisitive/Cur | .26 | -.01 | .30 | **.80** | -.03 | .00 |
| Intelligent | -.01 | -.19 | .09 | **.70** | -.08 | **.50** |
| Inventive | .28 | .03 | .20 | **.76** | .12 | .14 |
| Affect/Friendly | -.28 | -.03 | .27 | **.61** | **.41** | -.11 |
| Persistent | **.46** | -.34 | .10 | **.54** | .11 | .15 |
| Solitary | -.18 | .16 | -**.77** | -.18 | -.29 | .08 |
| Active | .26 | .09 | **.72** | **.47** | -.10 | .12 |
| Depressed | .03 | .32 | **-.76** | -.13 | .02 | .01 |
| Playful | .20 | .06 | **.67** | **.58** | -.06 | -.09 |
| Sexual | .29 | -.07 | **.65** | .01 | .30 | .22 |
| Calm | **-.57** | -.06 | **-.50** | .02 | .37 | -.10 |
| Relaxed | **-.44** | **.48** | **-.46** | .05 | .31 | -.15 |
| Excitable | **.56** | -.08 | **.49** | .19 | -.22 | .09 |
| Affiliative | .09 | .09 | **.53** | **.43** | **.49** | .02 |
| Protective | .15 | -.20 | .06 | -.08 | **.78** | .15 |
| Considerate | **-.44** | .16 | -.01 | .25 | **.63** | .14 |
| Autistic | **.42** | .36 | -.22 | .05 | -.03 | -.28 |
| Self-caring | .05 | .06 | .20 | .31 | .32 | **.55** |
| Methodical | .16 | **-.44** | -.26 | .30 | .33 | **.54** |

*Note.* **Bold** numbers have an eigenvalue greater than .40 for that factor. Underlined numbers have the highest loadings on that factor.

**SI 2.3**

**Age-category analyses**

**Supplementary Table 4:** Means for each factor for T1 and T2, and the T2-T1 difference, by age category.

|  | **Juvenile-adult** | | | **Adolescent-adult** | | | **Remained adult** | | |
| --- | --- | --- | --- | --- | --- | --- | --- | --- | --- |
| **Factor** | **T1** | **T2** | **T2-T1** | **T1** | **T2** | **T2-T1** | **T1** | **T2** | **T2-T1** |
| **Agreeableness** | 4.25 | 3.92 | **-.033** | 4.32 | 3.99 | **-.033** | 4.33 | 4.23 | **-0.10** |
| **Dominance** | 3.92 | 3.91 | **-0.01** | 4.26 | 4.68 | **0.42** | 4.17 | 4.72 | **0.55** |
| **Extraversion** | 5.34 | 5.03 | **-0.31** | 4.8 | 4.66 | **-0.14** | 4.66 | 4.66 | **0.00** |
| **Methodical** | 4.63 | 4.56 | **-0.07** | 4.67 | 4.55 | **-0.12** | 4.63 | 4.68 | **0.05** |
| **Openness** | 5.03 | 4.52 | **-0.51** | 4.62 | 4.61 | **-0.01** | 4.76 | 4.89 | **0.12** |
| **Reactivity/**  **Undependability** | 4.15 | 3.44 | **-0.71** | 4.05 | 3.42 | **-0.63** | 3.75 | 3.25 | **-0.50** |

**Supplementary Table 5:** Number of individuals changing over the study period for each factor, as measured by the RCI, by age category.

|  | **Juvenile-adult**  **N = 4** | **Adolescent-adult**  **N = 20** | **Remained adult**  **N = 26** |
| --- | --- | --- | --- |
| **Agreeableness** | 0 | 3 | 3 |
| **Dominance** | 0 | 3 | 5 |
| **Extraversion** | 0 | 0 | 1 |
| **Methodical** | 0 | 2 | 1 |
| **Openness** | 2 | 4 | 2 |
| **Reactivity/**  **Undependability** | 3 | 13 | 13 |

**SI 3 Additional discussion**

**Supplementary Tables 6 and 7:** A comparison of the six factors used in the study by King et al. (2008) (SI Table 4), and in the present study (SI Table 5). In both tables, (-ve) denotes negative loadings.

**SI Table 6: The six personality factors with their corresponding traits used in King et al. (2008)**

| **Agreeableness** | **Conscientiousness** | **Dominance** | **Extraversion** | **Openness** | **Neuroticism** |
| --- | --- | --- | --- | --- | --- |
| Gentle | Predictable | Bullying | Active | Inventive | Excitable |
| Helpful | Aggressive (-ve) | Decisive | Affectionate | Inquisitive | Stable (-ve) |
| Protective | Defiant (-ve) | Dominant | Friendly |  | Unemotional (-ve) |
| Sensitive | Disorganised (-ve) | Independent | Imitative |  |  |
| Sympathetic | Erratic (-ve) | Intelligent | Playful |  |  |
|  | Impulsive (-ve) | Persistent | Sociable |  |  |
|  | Irritable (-ve) | Stingy | Depressed (-ve) |  |  |
|  | Jealous (-ve) | Cautious (-ve) | Lazy (-ve) |  |  |
|  | Reckless (-ve) | Dependent (-ve) | Solitary (-ve) |  |  |
|  |  | Fearful (-ve) |  |  |  |
|  |  | Submissive (-ve) |  |  |  |
|  |  | Timid (-ve) |  |  |  |

**SI Table 7: The six personality factors with their corresponding traits used in the present study (taken from Table 1, main text).**

*Research on human personality stability*

There are very few longitudinal studies of personality in humans are based on non-Western samples. Populations can differ dramatically in physical and social environments (Henrich, Heine, & Norenzayan, 2010), and, although longitudinal research on human personality stability outside of Western samples is very limited, there is some evidence that certain traits may exhibit different longitudinal trajectories in non-Western populations compared to Western populations (see Costa, McCrae, & Löckenhoff, 2019 for a recent discussion on variance and invariance of personality across age groups and cultures). Accordingly, the basis of our human-chimpanzee comparisons are limited to data on western populations of humans and there is a need for more longitudinal research on human personality stability in non-Western populations to ascertain whether such comparisons are generalizable beyond western populations.

*Comparisons between human and chimpanzee personality stability*

Personality development in both humans and chimpanzees is thought to reflect sexual selection pressures and, at least in part, social factors or life events (King, Weiss, & Sisco, 2008; Srivastava, John, Gosling, & Potter, 2003). Social species such as chimpanzees face many important socially related issues including status competition, hierarchical conflicts and cooperation, and in fission-fusion societies these events are often dynamic in nature. Our findings that chimpanzee males are more dominant and extraverted, but less agreeable than females likely reflects the fact that males invest more effort than females to increase their social status, while investing relatively little in their offspring (Weiss & King, 2015). This corresponds with several findings that human males are rated as more assertive, risk-prone, active and less considerate and affiliative than females (Chapman, Duberstein, Sörensen, & Lyness, 2007; Costa, Terracciano, & McCrae, 2001; Weisberg, Deyoung, & Hirsh, 2011). Additionally, chimpanzees here were rated as less reactive/undependable over time. While this factor is specific to the instrument used in this study, it incorporates several facets of neuroticism as measured in humans (jealousy, manipulative, excitable, temperamental and (negatively loaded) calm), and thus, could potentially be seen as convergent with findings that humans decline in neuroticism with age (Roberts & DelVecchio, 2000; Srivastava et al., 2003) – though further work would be needed to verify this suggestion.

The majority of the subjects in this study were also captive born and raised at the facility, and there were no offspring born throughout the study period. In contrast, in wild chimpanzees, groups engage in fission fusion dynamics, female chimpanzees often migrate to new groups at adolescence and breeding provides a mixture of generations of chimpanzees (Aureli et al., 2008; Nishida, Hiraiwa-Hasegawa, Hasegawa, & Takahata, 2010). There is tentative evidence that mother-reared and nursery-reared chimpanzees differ on fine-grained aspects of personality dimensions (Latzman, Freeman, Schapiro, & Hopkins, 2015), and Weiss et al. (Weiss, King, & Hopkins, 2007) found some differences (as well as consistencies) in personality ratings when comparing chimpanzees from laboratory and zoo settings. There is also some evidence that humans display cultural differences in personality dimensions, including on measures of the Big Five traits (Carlo, Knight, Roesch, Opal, & Davis, 2013; Schmitt, Allik, McCrae, & Benet-Martínez, 2007; Smaldino, Lukaszewski, von Rueden, & Gurven, 2019). Although chimpanzees display arguably the widest repertoire of cultural behaviours in the animal kingdom and indeed the NCCC chimpanzees show group differences in experimentally seeded behaviours (Hopper et al., 2007; Luncz & Boesch, 2014; Whiten, 2017; Whiten et al., 1999, 2007), we know of no study examining whether chimpanzees display population differences in personality that could be attributable to cultural differences, these studies reinforces the importance of considering environmental and cultural factors in any comparisons with populations from different sites (also see Leavens, Bard, & Hopkins, 2010). Future research could also examine whether different chimpanzee groups (with large sample sizes) who inhabit the same environment display group level personality differences, as has been doing with extractive foraging (Rawlings, Davila-Ross, & Boysen, 2014) and social behaviours (van Leeuwen, Cronin, & Haun, 2014; van Leeuwen, Cronin, Haun, Mundry, & Bodamer, 2012).

Additionally, it is also possible that having offspring may impact the parents’ (mothers) personality over long time periods. However, given that breeding in captive chimpanzee populations has been banned in the US (and several other countries, Knight, 2008) since 2007, there is little data on how the birth of offspring may impact chimpanzee mother personality and there was no births during the study period (as noted above, there was no offspring born during the study period and the most recent birth at the NCCC was in 2002).

Finally, although personality here was measured using carestaff ratings, our findings may be informative for research in the stability of behavioural syndromes (correlated groups of behavioural traits), as personality often correlates with behavioural syndromes (Sih, Bell, & Johnson, 2004). Some recent studies have shown low correlations between the stability of individual personality traits and behavioural syndromes (Trnka, Samaš, & Grim, 2018; Wuerz & Krüger, 2015), potentially suggesting that stable behavioral syndromes may be grounded on less stable individual traits. An important avenue of future research, particularly with nonhuman primates and over longer time frames, is to verify and replicate these findings (Wuerz & Krüger, 2015).

**Supplementary References**

Aureli, F., Schaffner, C. M., Boesch, C., Bearder, S. K., Call, J., Chapman, C. a., … van Schaik, C. P. (2008). Fission‐Fusion Dynamics: New Research Frameworks. https://doi.org/10.1086/586708

Carlo, G., Knight, G. P., Roesch, S. C., Opal, D., & Davis, A. (2013). Personality across cultures: A critical analysis of Big Five research and current directions. In *APA handbook of multicultural psychology, Vol. 1: Theory and research.* (pp. 285–298). American Psychological Association. https://doi.org/10.1037/14189-015

Chapman, B. P., Duberstein, P. R., Sörensen, S., & Lyness, J. M. (2007). Gender differences in five factor model personality traits in an elderly cohort: extension of robust and surprising findings to an older generation. *Personality and Individual Differences*, *43*(06), 1594–1603. https://doi.org/10.1016/j.paid.2007.04.028

Costa, P. T., McCrae, R. R., & Löckenhoff, C. E. (2019). Personality Across the Life Span. *Annual Review of Psychology*, *70*(1), 423–448. https://doi.org/10.1146/annurev-psych-010418-103244

Costa, P. T., Terracciano, A., & McCrae, R. R. (2001). Gender differences in personality traits across cultures: Robust and surprising findings. *Journal of Personality and Social Psychology*, *81*(2), 322–331. https://doi.org/10.1037/0022-3514.81.2.322

Henrich, J., Heine, S. J., & Norenzayan, A. (2010). The weirdest people in the world? *Behavioral and Brain Sciences*, *33*(2–3), 61–83. https://doi.org/10.1017/S0140525X0999152X

Hopper, L. M., Spitari, A., Lambeth, S., Schapiro, S. J., Horner, V., & Whiten, A. (2007). Experimental studies of traditions and underlying transmission processes in chimpanzees. *Animal Behaviour*, *73*(6), 1021–1032. https://doi.org/10.1016/j.anbehav.2006.07.016

King, J. E., Weiss, A., & Sisco, M. M. (2008). Aping Humans: Age and Sex Effects in Chimpanzee (Pan troglodytes) and Human (Homo sapiens) Personality. *Journal of Comparative Psychology*, *122*(4), 418–427. https://doi.org/10.1037/a0013125

Knight, A. (2008). The beginning of the end for chimpanzee experiments? *Philosophy, Ethics, and Humanities in Medicine*, *3*(1), 16. https://doi.org/10.1186/1747-5341-3-16

Koo, T. K., & Li, M. Y. (2016). A Guideline of Selecting and Reporting Intraclass Correlation Coefficients for Reliability Research. *Journal of Chiropractic Medicine*, *15*(2), 155–163. https://doi.org/10.1016/j.jcm.2016.02.012

Latzman, R. D., Freeman, H. D., Schapiro, S. J., & Hopkins, W. D. (2015). The contribution of genetics and early rearing experiences to hierarchical personality dimensions in chimpanzees (Pan troglodytes). *Journal of Personality and Social Psychology*, *109*(5), 889–900. https://doi.org/10.1037/pspp0000040

Leavens, D. A., Bard, K. A., & Hopkins, W. D. (2010). BIZARRE chimpanzees do not represent “the chimpanzee.” *Behavioral and Brain Sciences*, *33*(2–3), 100–101. https://doi.org/10.1017/S0140525X10000166

Luncz, L. V., & Boesch, C. (2014). Tradition over trend: Neighboring chimpanzee communities maintain differences in cultural behavior despite frequent immigration of adult females. *American Journal of Primatology*, *76*(7), 649–657. https://doi.org/10.1002/ajp.22259

Nishida, T., Hiraiwa-Hasegawa, M., Hasegawa, T., & Takahata, Y. (2010). Group Extinction and Female Transfer in Wild Chimpanzees in the Mahale National Park, Tanzania. *Zeitschrift Für Tierpsychologie*, *67*(1–4), 284–301. https://doi.org/10.1111/j.1439-0310.1985.tb01395.x

Rawlings, B., Davila-Ross, M., & Boysen, S. T. (2014). Semi-wild chimpanzees open hard-shelled fruits differently across communities. *Animal Cognition*, *17*(4), 891–899. https://doi.org/10.1007/s10071-013-0722-z

Roberts, B. W., & DelVecchio, W. F. (2000). The rank-order consistency of personality traits from childhood to old age: A quantitative review of longitudinal studies. *Psychological Bulletin*, *126*(1), 3–25. https://doi.org/10.1037/0033-2909.126.1.3

Schmitt, D. P., Allik, J., McCrae, R. R., & Benet-Martínez, V. (2007). The Geographic Distribution of Big Five Personality Traits. *Journal of Cross-Cultural Psychology*, *38*(2), 173–212. https://doi.org/10.1177/0022022106297299

Sih, A., Bell, A., & Johnson, J. C. (2004). Behavioral syndromes: an ecological and evolutionary overview. *Trends in Ecology & Evolution*, *19*(7), 372–378. https://doi.org/10.1016/j.tree.2004.04.009

Smaldino, P. E., Lukaszewski, A., von Rueden, C., & Gurven, M. (2019). Niche diversity can explain cross-cultural differences in personality structure. *Nature Human Behaviour*, *3*(12), 1276–1283. https://doi.org/10.1038/s41562-019-0730-3

Srivastava, S., John, O. P., Gosling, S. D., & Potter, J. (2003). Development of personality in early and middle adulthood: set like plaster or persistent change? *Journal of Personality and Social Psychology*, *84*(5), 1041–1053. https://doi.org/10.1037/0022-3514.84.5.1041

Trnka, A., Samaš, P., & Grim, T. (2018). Stability of a behavioural syndrome vs. plasticity in individual behaviours over the breeding cycle: Ultimate and proximate explanations. *Behavioural Processes*, *153*, 100–106. https://doi.org/10.1016/j.beproc.2018.06.003

van Leeuwen, E. J. C., Cronin, K. A., & Haun, D. (2014). A group-specific arbitrary tradition in chimpanzees (Pan troglodytes). *Animal Cognition*, *17*(6), 1421–1425. https://doi.org/10.1007/s10071-014-0766-8

van Leeuwen, E. J. C., Cronin, K., Haun, D. B. M., Mundry, R., & Bodamer, M. D. (2012). Neighbouring chimpanzee communities show different preferences in social grooming behaviour. *Proceedings of the Royal Society B: Biological Sciences*, *279*(1746), 4362–4367. https://doi.org/10.1098/rspb.2012.1543

Weisberg, Y. J., Deyoung, C. G., & Hirsh, J. B. (2011). Gender differences in personality across the ten aspects of the Big Five. *Frontiers in Psychology*, *2*, 178. https://doi.org/10.3389/fpsyg.2011.00178

Weiss, A., & King, J. E. (2015). Great ape origins of personality maturation and sex differences: A study of orangutans and chimpanzees. *Journal of Personality and Social Psychology*, *108*(4), 648–664. https://doi.org/10.1037/pspp0000022

Weiss, A., King, J. E., & Hopkins, W. D. (2007). A cross-setting study of chimpanzee (Pan troglodytes) personality structure and development: zoological parks and Yerkes National Primate Research Center. *American Journal of Primatology*, *69*(11), 1264–1277. https://doi.org/10.1002/ajp.20428

Whiten, A. (2017). Social learning and culture in child and chimpanzee. *Annual Review of Psychology*, *68*(1), 129–154. https://doi.org/10.1146/annurev-psych-010416-044108

Whiten, A., Goodall, J., McGrew, W. C., Nishida, T., Reynolds, V., Sugiyama, Y., … Boesch, C. (1999). Cultures in chimpanzees. *Nature*, *399*(6737), 682–685. https://doi.org/10.1038/21415

Whiten, A., Spiteri, A., Horner, V., Bonnie, K. E., Lambeth, S. P., Schapiro, S. J., & de Waal, F. B. M. (2007). Transmission of multiple traditions within and between chimpanzee groups. *Current Biology*, *17*(12), 1038–1043. https://doi.org/10.1016/j.cub.2007.05.031

Wuerz, Y., & Krüger, O. (2015). Personality over ontogeny in zebra finches: Long-term repeatable traits but unstable behavioural syndromes. *Frontiers in Zoology*, *12*(1), S9. https://doi.org/10.1186/1742-9994-12-S1-S9
